# Dramatic Improvement of Subcutaneous Calcinosis by Intermittent, High-Dose Etidronate plus Cimetidine in a Patient with Juvenile Dermatomyositis

**DOI:** 10.1155/2015/817592

**Published:** 2015-11-12

**Authors:** Takayuki Wakabayashi, Noriko Sasaki, Naofumi Chinen, Yasuo Suzuki

**Affiliations:** ^1^Department of Rheumatology, Tokai University Hachioji Hospital, 1838 Ishikawamachi, Hachioji 192-0032, Japan; ^2^Division of Rheumatology, Department of Internal Medicine, Tokai University School of Medicine, 143 Shimokasuya, Isehara 259-1193, Japan

## Abstract

A 17-year-old boy with juvenile dermatomyositis presented with typical skin symptoms, mild myositis, and bilateral lower limb calcinosis. His skin and muscle symptoms responded to treatment with prednisolone and azathioprine. However, calcinosis did not improve, and the patient had a limited range of knee joint motion and resultant disturbance of daily activities. Cimetidine was combined with intermittent administration of high-dose etidronate, leading to marked improvement of both subcutaneous and muscular calcinosis with no skeletal adverse reactions during a long treatment period exceeding 5 years. As a result, the range of knee joint motion has increased and performance of daily activities has improved.

## 1. Introduction

Calcinosis occurs in various rheumatic diseases, such as systemic lupus erythematosus, scleroderma, and dermatomyositis [1]. Calcinosis is rare in adult dermatomyositis but is common in juvenile dermatomyositis, occurring in approximately 40% of patients [2]. Although various treatments have been tried for calcinosis and some have been reported to be effective, there is still no standard treatment modality. Here we report a patient with juvenile dermatomyositis in whom severe calcinosis was markedly improved by prolonged intermittent, high-dose administration of etidronate plus cimetidine.

## 2. Case Presentation

This patient was a 17-year-old boy who developed facial erythema and calcinosis around the left knee and femoral region at the age of 15 in 2001. He had undergone extensive resection of subcutaneous calcinosis within the year. In January 2003, he was referred to our hospital because of pain in the right knee and femoral region and skin rash on the face, the anterior chest, and the upper limbs. Physical examination revealed erythema of the forehead and buccal region, a V sign, a shawl sign, nail bed angiitis, and Gottron's papules. Although limb muscle weakness and elevation of muscle enzymes were slight, juvenile dermatomyositis was diagnosed because plain and contrast-enhanced MRI of the right femoral region revealed muscular atrophy, increased signal intensity on fat-suppression T2-weighted images, and enhancement by Gd-DTPA. There was prominent induration due to subcutaneous calcinosis around the right knee and femoral region, and the range of knee joint motion was limited.

**Figure 1:**
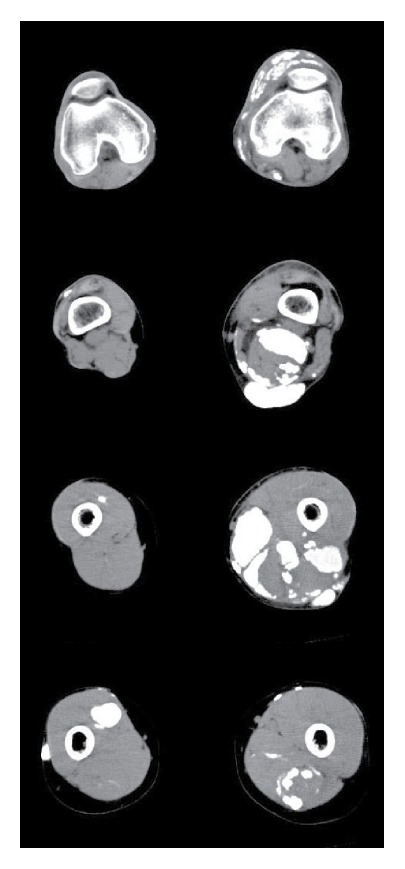
Before intermittent, high-dose etidronate therapy. Severe calcinosis is noted in the subcutaneous tissue and muscles of both lower limbs.

To treat juvenile dermatomyositis, administration of prednisolone was started at a dose of 20 mg/day. Then azathioprine was added at a dose of 100 mg/day for skin ulcers that developed during prednisolone treatment. Cimetidine was also administered for his subcutaneous calcinosis. Although his erythema, skin ulcers, and muscular symptoms tended to improve with these medications, the range of knee joint motion was still restricted and this interfered with his daily activities. In September 2009, CT scans of the lower limbs revealed extensive tumorous/nodular and diffuse lesions of calcinosis in the subcutaneous tissue and muscles extending from the thigh to the knee joint in both lower limbs (Figure 1).

**Figure 2:**
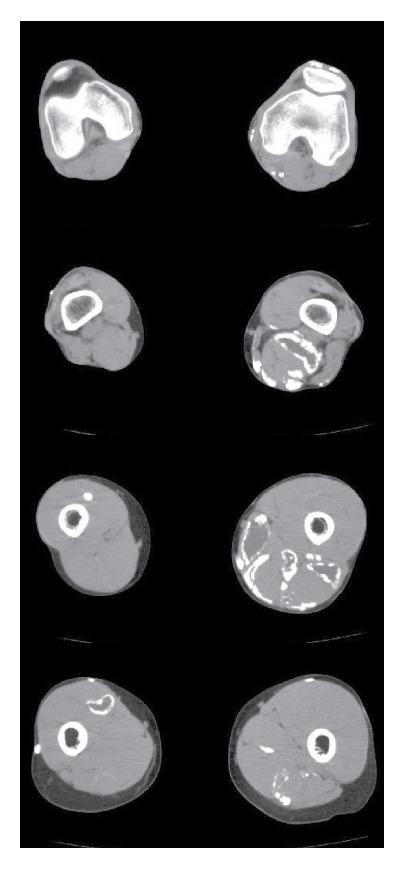
After 5 years of intermittent, high-dose etidronate therapy. Calcinosis shows marked improvement.

Accordingly, intermittent, high-dose treatment with etidronate was added for the calcinosis. Etidronate was administered at a dose of 800 mg/day for 3 months and then was discontinued for 6 months, with this intermittent treatment schedule being continued over 5 years. As a result, induration of the lower limb due to calcinosis resolved gradually, and the range of knee joint motion also increased. In August 2014, repeat CT of the lower limbs revealed a dramatic decrease of subcutaneous and muscular calcinosis (Figure 2).

At present (March 2015), the patient has no disturbance of daily activities. There have been no appreciable adverse reactions to etidronate therapy. The dose of prednisolone has been decreased to 5 mg/day and azathioprine has been discontinued without aggravation of skin or muscular symptoms.

## 3. Discussion

In this patient with juvenile dermatomyositis, extensive calcinosis showed marked improvement due to prolonged treatment with cimetidine and intermittent, high-dose etidronate. Electromyography and muscle biopsy were not done in this patient because he had diagnostic skin lesions and MRI findings. Instead, the diagnosis of juvenile dermatomyositis was based on the presence of weakness of proximal muscles and the elevation of myogenic enzymes in addition to typical skin symptoms and MRI findings. Although his skin and muscle symptoms responded to treatment with prednisolone and azathioprine, extensive calcinosis of the lower limbs did not show satisfactory improvement, resulting in persistence of a limited range of knee joint motion and disturbance of daily activities. Calcinosis is not rare in patients with juvenile dermatomyositis. While the pathogenesis has not been elucidated completely, it has been suggested that the onset of calcinosis is correlated with the disease duration and the length of disease activity [3, 4]. The low dose of prednisolone used in this patient may have contributed to progression of calcinosis. It has also been reported that macrophages are involved, along with cytokines such as IL-6, IL-1*β*, and TNF*α* [5]. There seems to be little likelihood of calcinosis resolving spontaneously. Because this patient had already undergone resection of calcinosis, we considered that active dermatomyositis had persisted for a long period since that operation until he attended our hospital.

Reports have been published concerning the treatment of calcinosis with colchicine, probenecid [6, 7], aluminium hydroxide [8], diltiazem [9, 10], high-dose immunoglobulin therapy [11], bisphosphonate preparations, and anti-TNF*α* preparations [12], but there is still no established regimen for this condition. 

Bisphosphonates inhibit the activity of osteoclasts and suppress bone resorption [1, 5], and these drugs are used for the treatment of osteoporosis, Paget's disease of bone, bone metastasis of malignant tumors, hypercalcemia, and multiple myeloma. Etidronate inhibits bone resorption when administered at low doses, while it inhibits bone mineralization as well as bone resorption at high doses. The reported efficacy of etidronate for calcinosis in rheumatic diseases varies widely. Rabens and Bethune [13] reported that etidronate was effective for calcinosis in patients with scleroderma, while Metzger et al. [14] stated that it was ineffective for calcinosis associated with scleroderma and dermatomyositis. In the present patient with juvenile dermatomyositis, etidronate was extremely effective for calcinosis, but this might have been partly ascribable to coadministration of cimetidine which is also considered to inhibit calcinosis. There have been reports that aminobisphosphonates such as pamidronate [15, 16] and alendronate [5] are effective for calcinosis in patients with juvenile dermatomyositis, although these aminobisphosphonates have a different mechanism of action from etidronate.

Prolonged treatment with high-dose etidronate is associated with a risk of osteomalacia, which is one of the important adverse reactions to this drug.

Therefore, we monitored skeletal adverse reactions periodically by taking X-ray of bones and measuring vertebral and femoral bone mineral densities. However, no deleterious effect of etidronate on bones has been observed and our patient has been able to use etidronate according to an intermittent schedule for over 5 years.

In this patient with juvenile dermatomyositis, cimetidine combined with intermittent, high-dose administration of etidronate was effective for calcinosis. Thus, bisphosphonate therapy seems to be one of the useful options for calcinosis, although further studies in more patients will be needed to confirm its efficacy and safety.
